# A novel d-xylose isomerase from the gut of the wood feeding beetle *Odontotaenius disjunctus* efficiently expressed in *Saccharomyces cerevisiae*

**DOI:** 10.1038/s41598-021-83937-z

**Published:** 2021-02-26

**Authors:** Paulo César Silva, Javier A. Ceja-Navarro, Flávio Azevedo, Ulas Karaoz, Eoin L. Brodie, Björn Johansson

**Affiliations:** 1 CBMA - Center of Molecular and Environmental Biology, University of Minho, Campus de Gualtar, 4710-057 Braga, Portugal; 2 Biological Systems and Engineering, Lawrence Berkeley National Laboratory, Berkeley, CA USA; 3 Institute for Biodiversity Science and Sustainability, California Academy of Sciences, San Francisco, CA USA; 4 Earth and Environmental Sciences, Lawrence Berkeley National Laboratory, Berkeley, CA USA; 5 Department of Environmental Science, Policy and Management, University of California, Berkeley, CA USA

**Keywords:** Biotechnology, Microbiology, Molecular biology

## Abstract

Carbohydrate rich substrates such as lignocellulosic hydrolysates remain one of the primary sources of potentially renewable fuel and bulk chemicals. The pentose sugar d-xylose is often present in significant amounts along with hexoses. *Saccharomyces cerevisiae* can acquire the ability to metabolize d-xylose through expression of heterologous d-xylose isomerase (XI). This enzyme is notoriously difficult to express in *S. cerevisiae* and only fourteen XIs have been reported to be active so far. We cloned a new d-xylose isomerase derived from microorganisms in the gut of the wood-feeding beetle *Odontotaenius disjunctus*. Although somewhat homologous to the XI from *Piromyces* sp. E2, the new gene was identified as bacterial in origin and the host as a *Parabacteroides* sp. Expression of the new XI in *S. cerevisiae* resulted in faster aerobic growth than the XI from *Piromyces* on d-xylose media. The d-xylose isomerization rate conferred by the new XI was also 72% higher, while absolute xylitol production was identical in both strains. Interestingly, increasing concentrations of xylitol (up to 8 g L^−1^) appeared not to inhibit d-xylose consumption. The newly described XI displayed 2.6 times higher specific activity, 37% lower K_M_ for d-xylose, and exhibited higher activity over a broader temperature range, retaining 51% of maximal activity at 30 °C compared with only 29% activity for the *Piromyces* XI.

## Introduction

Lignocellulosic material continues to be the most promising renewable raw material for the production of sustainable fuels and fine chemicals^1^. Xylan is the second most abundant biopolymer on earth which contains mostly the pentose sugar d-xylose^2^. Baker’s yeast or *Saccharomyces cerevisiae* is the preferred organism for industrial transformation of sugars derived from lignocellulose due to innate resistance to fermentation inhibitors^3^. Expression of heterologous pathways are necessary for d-xylose utilization as it is not metabolized naturally by *S. cerevisiae*. d-xylose metabolism remains a metabolic bottleneck in *S. cerevisiae* despite the development of several types of pathways for the consumption of this sugar^4^.

d-xylose metabolic pathways can be classified into two main categories, d-xylose reductase/xylitol dehydrogenase (XR/XDH) and d-xylose isomerase (XI). The XR/XDH pathway converts d-xylose to xylitol by reduction with NADPH or NADH followed by an oxidation with NAD^+^ to xylulose in an overall redox neutral process. Alternatively, the same reaction is carried out by a single XI enzyme without cofactors. The XR/XDH pathway is mainly found in fungi while the XI pathway is common in prokaryotes. The currently most promising d-xylose metabolic pathways are based on the prokaryotic XI route. The reason for this is that although the overall reaction is redox neutral, the XR/XDH pathway suffers from a NAD(P)H cofactor imbalance that has proven hard to remedy^5–7^. However, the XI pathway suffers from low capacity and inhibition by xylitol^8^. Another issue is that the XI is rather difficult to express heterologously in yeast. Several unsuccessful attempts have been made to express XIs, such as those from *Escherichia coli*^9,10^* Bacillus subtilis, Actinoplanes missouriensis*^11^, *Lactobacillus pentosus*^12^ and *Clostridium thermosulfurogenes* (renamed as *Thermoanaerobacterium thermosulfurigenes*)^13^. The first successfully expressed XI was a thermostable enzyme from *Thermus thermophilus*^14^ followed by an XI originating from the fungus *Piromyces* sp.^15^.

Only fourteen different XIs with characterized kinetic parameters have been reported to actively express in *S. cerevisiae* (Table 1). Interestingly, the fungal XIs (Table 1, entry #2 and #3) both come from the same division (*Neocallimastigomycota*). These fungi are known for possessing genes originating via lateral gene transfer. Their XIs are of prokaryotic origin and have been recently incorporated in evolutionary terms^16^. These fungal isomerases were isolated from fungi residing in the intestines of herbivorous mammals, an ecosystem typically poised at 37 °C and with dense populations of co-occurring bacteria. Interestingly, only four XIs have been isolated directly from metagenomes and subsequently expressed in *S. cerevisiae* (Table 1, #5 and #6, #12, #14). Two XIs were derived from soil samples, one using degenerate primers and another by functional metagenomic screening using *E. coli* with a nonfunctional *xylA*^17^. An additional XI was amplified from bovine rumen contents using degenerate primers targeting relatively conserved XI specific sequences^18^, and yet another derived from protists in the hindgut of the termite *Reticulitermes speratus* using a similar approach^19^.

**Table 1:** Literature data on d-xylose isomerase enzymes with characterized kinetic properties that were actively expressed in *Saccharomyces cerevisiae*.

#	Source	Type	Codon optimization	K_M_	Vmax (U mg^−1^)	References
1	*Thermus thermophilus*	Prokaryote, Gram−	No	NA	1.0^(a)^	^14^
2	*Piromyces* sp. E2	Eukaryote	No	20	1.1	^15^
			Yes	49.85	0.0538	^8^
			Yes^(b)^	50.18	0.083	^22^
			No	51	0.25	^23^
			Yes	3.9^(c)^	2.68^(c)^	^24^
			Yes	14.90	0.0103	^19^
			Yes	6.2^(c)^	8.3^(c)^	^25^
			Yes	30.76	0.0112	This work
3	*Orpinomyces* sp. ukk1	Eukaryote	No	NA	1.91	^26^
4	*Clostridium phytofermentans*	Prokaryote, Gram+	Yes	66.01	0.0344	^8^
			Yes^(b)^	37.1	0.107	^27^
5	Soil—xym1 (unspecified)	Prokaryote	No	NA	0.33	^17^
6	Soil—xym2 (unspecified)	Prokaryote	No	NA	0.20	^17^
7	*Bacteroides stercoris*	Prokaryote, Gram−	No	54.03	NA	^28^
8	*Ruminococcus flavefaciens*	Prokaryote, Gram+	Yes^(d)^	66.7	1.41	^29^
9	*Prevotella ruminicola*	Prokaryote, Gram−	Yes^(e)^	34	0.81	^23^
10	*Burkholderia cenocepacia*	Prokaryote, Gram−	No	NA	~ 0.037	^30^
			No	17.08^(c)^	44.97^(c)^	^31^
11	*Bacteroides vulgatus*	Prokaryote, Gram−	No	NA	~ 1.25	^32^
12	Bovine rumen (unspecified)	Prokaryote	No^(e)^	16.8	1.31	^18^
13	*Sorangium cellulosum*	Prokaryote, Gram-	No^(e)^	17.2	0.35	^18^
14	Termite gut (unspecified)	Eukaryote	Yes	10.52	0.0074	^19^
15	Passalid beetle gut—8054_2 (unspecified)	Prokaryote	Yes	19.27	0.0296	This work

Kinetic parameters were determined directly from cell lysates or otherwise stated.

*NA* not available.

^a^Determined by measuring the conversion of fructose to glucose.

^b^And also random mutagenesis.

^c^Determined from purified enzymes.

^d^And also site-directed mutagenesis and modifications 5′-end of the gene.

^e^And also evolutionary adaptation.

An alternative and less biased approach to retrieving novel XIs is through the assembly of shotgun metagenomic sequences from environments thought to be enriched in d-xylose utilization capacity. Following gene prediction, *in-silico* translation and annotation against appropriate databases, a subset of XI genes may then be optimized computationally for a specific host, and then synthesized in-vitro. This strategy avoids the bias associated with PCR amplification and is expected to broaden the discovery of enzymes with novel properties. Using this approach, we have identified 182 putative XIs (coded by *xylA* genes) from the microbial metagenome in the gut of a passalid beetle (*Odontotaenius disjunctus*)^20^, known to subsist on d-xylose rich hardwoods, and to harbor d-xylose utilizing yeasts^21^*.* We codon optimized and synthesized three XIs, one of which expressed successfully in *S. cerevisiae*, highlighting that direct metagenome reconstruction combined with in-vitro gene synthesis is a viable approach for the identification of novel XI genes for expression in *S. cerevisiae*. In the present work, this new XI was characterized and compared to the homolog from *Piromyces* sp. The d-xylose isomerization capacity of the expressed XIs were evaluated in-vivo by yeast growth and by d-xylose conversion rates. Enzyme kinetics and optimal temperature were determined in-vitro using cell extracts. Additionally, a phylogenetic analysis of the XIs that have been expressed efficiently or that could not be expressed in yeast was carried out to interpret the role of evolutionary relationships in the expression of these enzymes.

## Results

### Identification of XI genes from metagenomic information

The metagenome of the wood-feeding beetle *O. disjunctus,* previously reconstructed by our team members^20^, was screened for the detection of d-xylose isomerases against the pfamA database. A total of 182 putative XI sequences were detected and phylogenetically placed together with the XI of *Piromyces* (see Fig. S1, Supplementary Information). Based on their phylogenetic relatedness, 3 metagenome-derived XI sequences were selected (labeled 8054_2, 15405_2, and 1362_6 in Fig. S1) and further characterized using the SWISS-MODEL workspace^33^. Top SWISS-MODEL templates for each of the target amino acid sequences corresponded to d-xylose isomerases for which crystal structures are available (see Table S1, Supplementary Information). Sequences 1362_6 and 14450_2 had 55.9% and 56.7% sequence homology with the XI of *Thermotoga neapolitana*, respectively, while 8054_2 had 82.5% homology with the XI of *Bacteroides thetaiotaomicron*. Reconstructed structure-homology models for each sequence also showed high quality scores between target and template (GMQE) and agreement between model and structure (QMEAN) (see Table S1, Supplementary Information). Based on these results, we proceeded to synthesize codon-optimized versions of these *xylA* genes for expression in *S. cerevisiae.*

**Figure 1:**
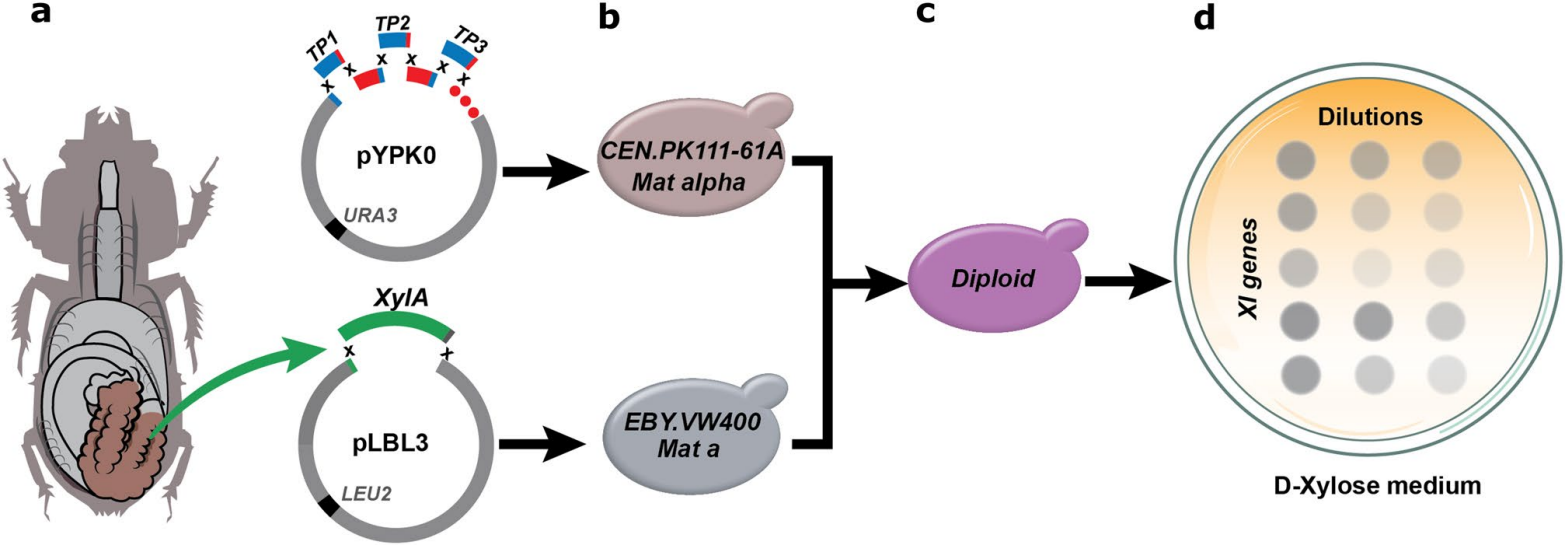
Strategy for the screening of d-xylose isomerases (XIs) in *Saccharomyces cerevisiae*. (**a**) Putative *xylA* genes from the microbial metagenome in the gut of a passalid beetle (*Odontotaenius disjunctus*) were codon-optimized, synthesized and cloned in the plasmid pLBL3; the partial d-xylose pathway (*TKL1*, *TAL1*, *RPE1*, *RKI1*, *XKS1*, and Gxf1) was constructed in the plasmid pYPK0 under the control of different terminators/promoters (TP). (**b**) The plasmids containing *xylA* genes (pLBL3_XI) were carried by the strain EBY.VW400 and the plasmid containing the partial d-xylose pathway (pYPK0_XTTRRG) was carried by the strain CEN.PK111-61A. (**c**) Each EBY.VW4000 strain clone was mated with the CEN.PK111-61A strain. (**d**) Candidate XI enzymes were functionally screened by scoring yeast growth on solid media with d-xylose as the sole or main carbon source.

The codon-optimized synthetic genes corresponding to the sequences of 8054_2, 15405_2, 1362_6 and *Piromyces* sp. *xylA* were cloned into the plasmid pLBL3 under the control of a constitutive TEF promoter. Plasmid pLBL3_XR/XDH expressing *Scheffersomyces stipitis* d-xylose reductase (XR) and d-xylitol dehydrogenase (XDH) genes (GenBank Gene ID: 4839234 and 4852013) corresponding to the initial fungal d-xylose metabolic pathway was also constructed. The resulting plasmids were transformed into *S. cerevisiae* EBY.VW4000. In parallel, a plasmid containing a partial d-xylose utilization pathway (*TKL1*, *TAL1*, *RPE1* and *RKI1*), xylulokinase (*XKS1*), and a d-xylose/glucose facilitator from *Candida intermedia* (Gxf1), was constructed using the yeast pathway kit^34^ in the strain CEN.PK111-61A (Fig. 1). Yeast strains containing d-xylose isomerase genes or XR/XDH pathway were mated to the strain with the partial d-xylose utilization pathway. The resulting diploid strains were plated on solid media with d-xylose as the sole carbon source. The strains expressing the XR/XDH gene pair, the *Piromyces* sp. *xylA,* and the 8054_2 gene produced growth clearly distinguishable from strains with the partial d-xylose utilization pathway and the empty pLBL3 vector. Strains expressing the 15405_2 and 1362_6 genes showed no discernible growth.

### Phylogenetic relationship among yeast (un)expressed XIs

**Figure 2:**
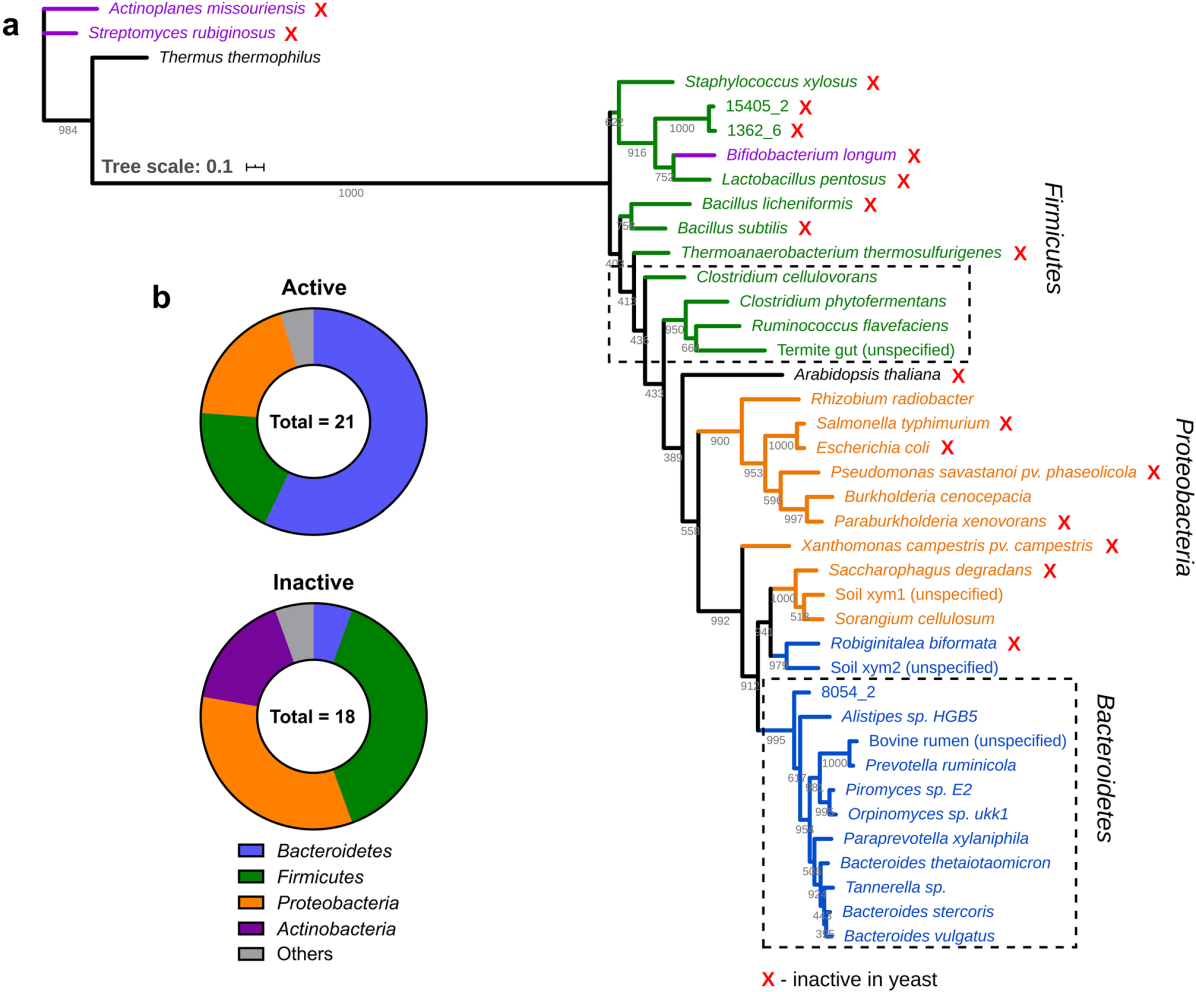
Phylogenetic analysis of the amino acid sequences of XIs that have been reported to actively express or not to express in *Saccharomyces cerevisiae*. Data from patents was not considered in the analysis. (**a**) In the phylogenetic tree, *Firmicutes* are colored green, *Proteobacteria* are colored orange, *Bacteroidetes* are colored blue, *Actinobacteria* are colored purple, and XIs from other sources are colored black. Dashed squares highlight two clusters of XIs belonging to *Clostridia* class (*Firmicutes*) and *Bacteroidia* class (*Bacteroidetes*) that expressed actively in yeast. (**b**) Ratio of active and inactive XIs expressed in yeast from the taxonomic groups presented in the panel A. GenBank accession numbers of the amino acid sequences are available in Table S2, Supplementary Information.

Phylogenetic analysis was used to further distinguish the successfully expressed from the unexpressed metagenome derived XIs and to complement the phylogenetic analysis from the SWISS-MODEL (Fig. 2a). For this purpose, we also included sequences of XIs for which activity has been reported in *S. cerevisiae* to evaluate any evolutionary relationships that might exist between successfully and unsuccessfully expressed enzymes. The sources of XIs included in the phylogenetic analysis were divided in three main prokaryotic phyla: *Firmicutes* (Fig. 2a—colored green), *Proteobacteria* (orange), and *Bacteroidetes* (blue); two other phyla that contain few representatives: *Actinobacteria* (purple; *Streptomyces rubiginosus*, *Actinoplanes missouriensis, Bifidobacterium longum*) and *Deinococcus-Thermus* (*Thermus thermophilus*); and one in *Plantae* (*Arabidopsis thaliana*). The XIs from the fungi *Piromyces* sp. and *Orpinomyces* sp. share 95% identity and were clustered within the *Bacteroidetes* phylum. *T. thermophilus* showed 55% and 59% identity with *A. missouriensis* and *S. rubiginosus*, respectively, but these three enzymes show very low identity with the other XIs (< 29%). XIs from metagenomic libraries were grouped within the prokaryotic phyla similar to previous reports^17–19^.

In the phylogenetic tree, 8054_2 XI was clustered with *Bacteroidetes* sharing 82% identity with *B. thetaiotaomicron*, and 78% with *Piromyces* sp. Sequence 8054_2 was previously reported as belonging to a metagenome assembled-genome (MAG) identified as *Parabacteroides* sp. which was highly abundant in the mostly anaerobic anterior hindgut of *O. disjunctus*^20^. A BLAST analysis also showed that 8054_2 XI shares the highest identity with XIs from *Bacteroidetes* phylum, particularly with the *Porphyromonadaceae* family of the *Bacteroidia* class. *Porphyromonadaceae* are commonly found in the gastrointestinal tract and oral cavity of animals^35^. The 8054_2 XI also shares high identity with the *Bacteroides* genus, such as *Bacteroides timonensis* (83%, accession number WP_044271094.1) that is an obligate anaerobe isolated from human feces^36^. The 15405_2 XI and 1362_6 XI share 94% identity and exhibit low similarity with the other XIs displayed in the tree. A BLAST analysis revealed high identity of both 15405_2 XI and 1362_6 XI with XIs from *Leuconostoc* genus of the *Firmicutes* phylum (85% and 86%; accession numbers WP_042252435.1, WP_004912802.1, respectively).

The expression success of XIs in yeast reveals a positive tendency towards the *Bacteroidetes* phylum (Fig. 2b). The majority of the reported active XIs in yeast are from *Bacteroidetes* (12 of 21), and most of the XIs from this phylum have been expressed successfully (12 of 13). The only XI belonging to *Bacteroidetes* that was not expressed in yeast is from *Robiginitalea biformata* that, coincidentally, is also the most dissimilar enzyme of this phylum, being positioned quite distant from the *Bacteroidetes* cluster. Inactive XIs (indicated by a red “X”) are comprised mostly of the other phyla: *Proteobacteria* (6 of 10), *Firmicutes* (7 of 11), and *Actinobacteria* (3 of 3). The XIs from *Proteobacteria* that have been successfully expressed in yeast (*Rhizobium radiobacter*, *Burkholderia cenocepacia*, *Sorangium cellulosum*) belong to different classes and do not share relevant ecological traits in the context of this work. The active XIs from *Firmicutes* (*Clostridium phytofermentans*, *Clostridium cellulovorans*, *Ruminococcus flavefaciens*) are from anaerobic cellulolytic organisms that belong to *Clostridia* class (Fig. 2a—*Firmicutes*’ dashed square). Similarly, actively expressed XIs clustered with *Bacteroidetes* are from anaerobic species that are commonly associated with the gastrointestinal tract of animals and belong to the same *Bacteroidia* class (*Bacteroidetes*’ dashed square), while the inactive XI belongs a different class (*Flavobacteriia*)^37^.

### Growth on d-xylose under aerobic conditions

**Figure 3:**
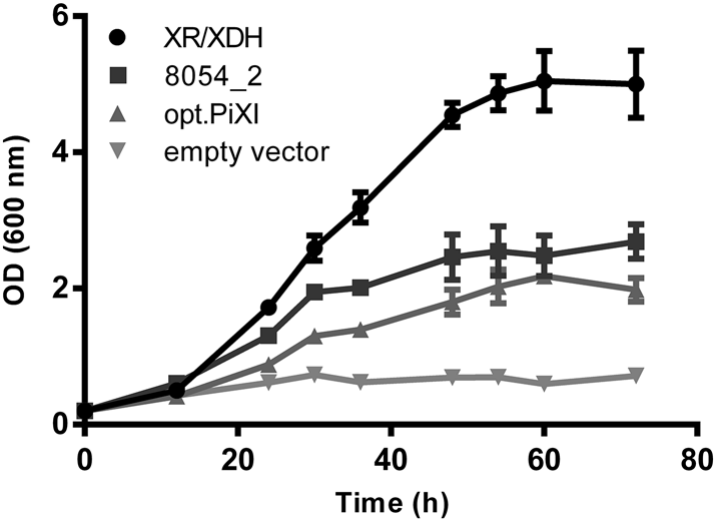
Aerobic growth of diploid *Saccharomyces cerevisiae* strains expressing 8054_2 XI, the codon-optimized XI from *Piromyces* sp. E2 (opt.PiXI), XR/XDH, or the empty vector. Cultures were grown in shaking-flasks on synthetic medium with 2% (w/v) d-xylose as the sole carbon source. Data points represent an average of 3 biological replicates with standard deviation indicated.

Growth performance of yeast diploids carrying the genes 8054_2, *xylA* from *Piromyces* sp. (opt.PiXI), and XR/XDH, or an empty vector was assessed in synthetic medium with 2% (w/v) d-xylose as the sole carbon source under aerobic conditions. The results of the growth are shown in Fig. 3. The negative control strain (Fig. 3—Empty vector), lacking XI or XR/XDH enzymes but expressing the partial d-xylose utilization pathway, did not proliferate. The cultures expressing enzymes that convert d-xylose to xylulose grew over time. The strain expressing XR/XDH proliferated at a maximal specific growth rate of 0.10 h^−1^, the strain expressing 8054_2 XI grew at 0.06 h^−1^, and the strain expressing opt.PiXI grew at 0.04 h^−1^. The XR/XDH pathway provided a considerably higher growth rate than the XI pathways as expected, and remarkably, 8054_2 XI conferred 50% faster growth in yeast than opt.PiXI.

### d-Xylose consumption under high cell density cultures

**Figure 4:**
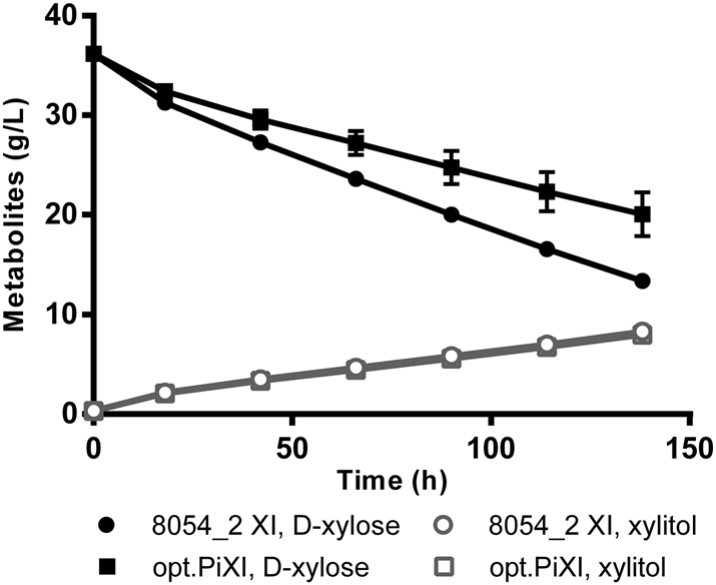
The d-xylose consumption by diploid *Saccharomyces cerevisiae* strains expressing 8054_2 XI or the codon-optimized XI from *Piromyces* sp. E2 (opt.PiXI). Cultures were grown under high cell density (10 g L^−1^ DCW) in shake flasks on synthetic medium with 4% (w/v) d-xylose as the sole carbon source. Data points represent an average of 3 biological replicates with standard deviation indicated.

Cultures were inoculated at a high cell density (~ 10 g L^−1^ DCW) in medium with 4% (w/v) d-xylose as the sole carbon source in shake-flasks at 1:20 liquid-to-air ratio. Both cultures started consuming d-xylose immediately after incubation and showed a steady d-xylose conversion until the end of the experiment (Fig. 4). The strain carrying 8054_2 XI consumed d-xylose at a rate of 0.015 g d-xylose h^−1^ g(DCW)^−1^, and the strain carrying opt.PiXI showed a consumption rate of 0.010 g d-xylose h^−1^ g(DCW)^−1^. The strain carrying XR/XDH depleted 96% of d-xylose after 18 h, corresponding to at least 0.187 g d-xylose h^−1^ g(DCW)^−1^ (see Fig. S2, Supplementary Information). The higher growth rate and d-xylose consumption conferred by the XR/XDH pathway compared to the XI pathway is consistent with previous work^38,39^. Xylitol was produced throughout the cultivation with a yield of 0.33 g xylitol g^−1^ d-xylose consumed for 8054_2 XI, and 0.47 g xylitol g^−1^ d-xylose consumed for opt.PiXI. Therefore, excluding d-xylose converted to xylitol rather than metabolized by the XI system, the rate of d-xylose isomerization was 0.0098 g d-xylose h^−1^ g(DCW)^−1^ for 8054_2 XI, and 0.0057 g d-xylose h^−1^ g(DCW)^−1^ for opt.PiXI*.* This represents a 72% higher d-xylose isomerization by 8054_2 XI than opt.PiXI. Absolute xylitol production was identical for both strains (~ 8 g L^−1^ after 138 h) due to the background activity of aldose reductases (such as *GRE3*) that reduce d-xylose to xylitol^40^. Xylitol has been shown to inhibit XI activity in-vitro^8,41^, although in our experiments, xylitol appears not to affect d-xylose consumption over time as it appears linear for the duration of the experiment for both strains.

### Kinetic parameters and optimal temperature

**Figure 5:**
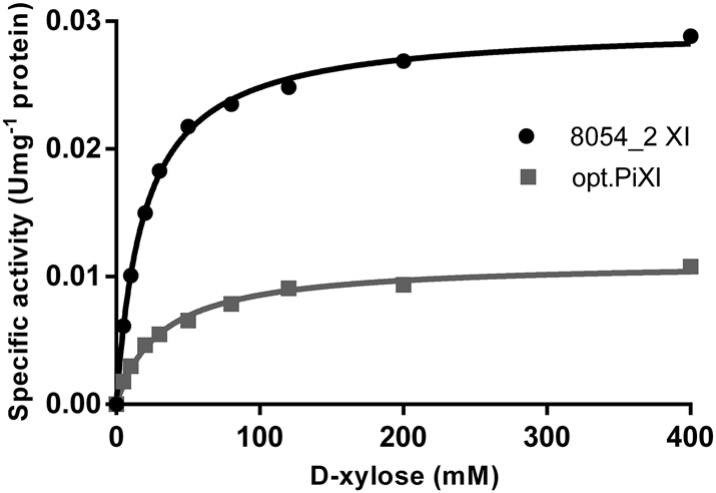
Enzymatic specific activity of the XI enzymes 8054_2 XI or the codon-optimized XI from *Piromyces* sp. E2 (opt.PiXI). Kinetic parameters were determined from a Michaelis–Menten plot adjusted to the experimental data (R squared > 0.99). Strains were grown in synthetic medium containing 2% (w/v) d-xylose until the exponential growth phase. Crude cell extracts were prepared and used immediately in the activity assays. Data points represent an average of 3 biological replicates.

**Figure 6:**
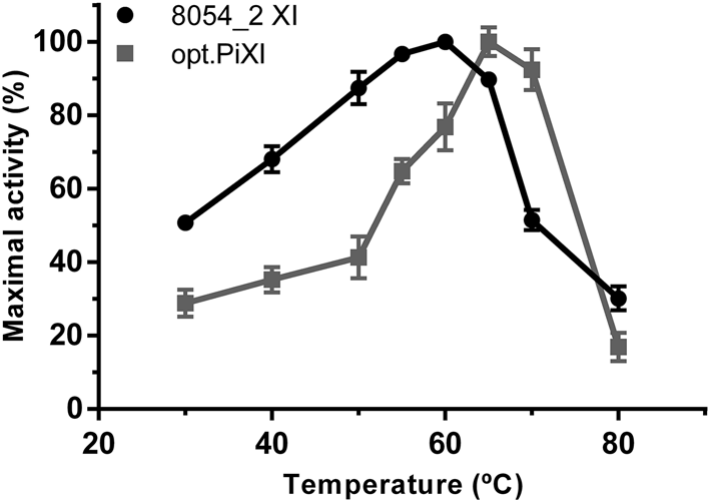
Relative enzymatic activity of XI enzymes 8054_2 XI and the codon-optimized XI from *Piromyces* sp. E2 (opt.PiXI) at different temperatures. Strains were grown in synthetic medium containing 2% (w/v) d-xylose until the exponential growth phase. Crude cell extracts were prepared and used immediately in the activity assays. Data points represent an average of 3 biological replicates with standard deviation indicated.

Kinetic properties of the enzymes 8054_2 XI and opt.PiXI were determined using crude cell extracts prepared from the recombinant diploid strains by a bead beating method. There seem to be no significant differences in the apparent protein expression level between both XIs in the cell extracts (see Fig. S3, Supplementary Information). A Michaelis–Menten curve was adjusted to the experimental values of both enzymes with close fit (Fig. 5). The extracts containing 8054_2 XI converted d-xylose at a maximal rate (V_max_) of 0.0296 U mg^−1^ total protein, and the extracts containing opt.PiXI showed a V_max_ of 0.0112 U mg^−1^ total protein. The 8054_2 XI exhibited a K_M_ for d-xylose of 19.27 ± 0.74 mM, and opt.PiXI exhibited a K_M_ of 30.76 ± 2.43 mM. Thus, the comparative analysis presented in this study indicates that 8054_2 XI operates at a 2.6 times higher V_max_ and with 37% lower K_M_ for d-xylose than opt.PiXI. We also assessed optimal enzyme temperature using a discontinuous colorimetric assay to measure relative activity of XIs in the cell extracts at different temperatures. The optimal temperatures of 8054_2 XI and opt.PiXI were approximately 60 °C and 65 °C, respectively (Fig. 6). Relative activity of both enzymes shows a steady increase from 30 °C, i.e., yeast optimum growth temperature, to the respective peak (100% activity) followed by a strong decrease to the lowest activity at 80 °C. Despite the similar thermophilic behavior, 8054_2 XI retains 51% of the maximal activity at 30 °C, while activity of opt.PiXI was reduced to 29%.

## Discussion

Novel metagenomic sequence data as well as sequence data in public databases represent a largely untapped resource for novel catalytic capability for future biotechnological processes. Making use of these data requires accurate assembly of sequence reads, gene identification followed by gene synthesis. In this work, we searched metagenomic data we derived from the hindgut of a wood-feeding beetle^20^ for similarity to known products of d-xylose isomerases genes, which depends on correctly translating metagenome sequence data *in-silico.* Gene synthesis remains a comparatively expensive approach relative to PCR amplification of DNA from complex extracts, with synthesis cost, time, and error rates increasing with sequence length. However, as well as eliminating PCR bias, an added benefit of gene synthesis is that its codon usage can be optimized for a specific host. Despite the expense, the use of such optimized genes is growing in popularity as evidenced by the twenty percent of sequences deposited at Addgene being composed of codon optimized genes^42^. Although some XI enzymes have been successfully expressed in *S. cerevisiae* allowing more or less efficient d-xylose metabolism, the screening for XIs with significant expression in yeast has proved difficult and often require a suite of optimizations^8,14,15,43^. Our strategy used here allowed direct selection of one very efficient XI from three synthesized candidate genes chosen from the three closest homologs to a known gene without any further evolutionary adaptation of the *S. cerevisiae* host. The separation of XI and the other genes necessary for efficient growth on d-xylose facilitate high-throughput functional screening which would enable testing of many more genes when gene synthesis costs are more permissive^44^.

d-xylose isomerases have been found in lignocellulosic material such as decomposing biomass, the gut of animals or their feces^15,17–19^ presumably due to the opportunity to consume d-xylose. Little is known about the relevance of phylogenetic relationship of different XIs for their efficient expression in *S. cerevisiae*, or whether certain microbial groups contain a pool of XIs with higher probability of being expressed in yeast. One of three of the XI enzymes screened in this work was successfully expressed (8054_2 XI) and clustered within the *Bacteroidetes* phylum (Fig. 2a). Curiously, all the previously assayed XIs from *Bacteroidia* and *Clostridia* (with exception to *T. thermosulfurigenes*) exhibited considerable activity in yeast (Fig. 2a—dashed squares). These classes of organisms have crucial ecological traits in common, such as an anaerobic metabolism and a remarkable capacity for degrading polysaccharides^37,45–47^. This indicates that *Bacteroidia* and *Clostridia*, particularly species residing in niches where active degradation of lignocellulosic biomass could be considered a selective pressure, may represent a valuable source of new XIs that express efficiently in yeast.

The growth rates on d-xylose presented in this work are comparable to others using non-evolved *S. cerevisiae* CEN.PK-based strains with identical genetic modifications. Comparatively, 8054_2 XI produced 50% higher growth rates and 72% higher d-xylose isomerization than the XI from *Piromyces* sp. under identical conditions, which indicates that the 8054_2 XI is an improved option for metabolic engineering of S. *cerevisiae* to transform d-xylose. Nevertheless, identical strains carrying the XI from *Piromyces* sp. have exhibited very distinct growth rates, which vary from 0.02 to 0.07 h^−1^^8,17,38^, or 0.21 to 0.22 h^−1^^24,43^. The growth rates conferred by the XI enzymes expressed in this work are closer to the first group of values. The second group is among the highest specific growth rates on d-xylose reported in literature for any XI or evolved strain^22,23,32,48,49^. This discrepancy may be attributed to a wide range of factors including unknown beneficial genetic modifications.

Xylitol has been identified as a competitive inhibitor of XIs^41^. Interestingly, contradictory results on xylitol inhibition have been reported. According to in-vitro assays, XI from *Piromyces* sp. is strongly inhibited in-vitro at a concentration of 50 mM xylitol, and an inhibition constant of 4.6 mM was determined^8^. Similarly, XI from *Bacteroides stercoris*, with an inhibition constant of 5.1 mM xylitol, showed a 50% decrease in activity at a concentration of 30 mM xylitol, although the fermentation rate only declined by less than 10%^28^. In our study, xylitol had no noticeable effect on d-xylose isomerization by either enzyme even at a concentration of approximately 50 mM (8 g L^−1^).

The kinetic parameters of the XI from *Piromyces* sp. reported in literature are very dissimilar, reflecting the vast experimentation with this enzyme (Table 1, entry #2). To overcome this limitation, in our study, 8054_2 XI and opt.PiXI were compared under identical conditions to eliminate this source of uncertainty. The 8054_2 XI showed significantly higher specific activity and affinity relative to opt.PiXI, both correlated with higher relative activity at 30 °C. As most of the XIs expressed in yeast are thermophilic, enzyme activity is dramatically reduced at industrial temperatures of fermentation that vary from 30 to 40 °C^50^. Only one yeast-expressed XI in the mesophilic range has been described to date^26^, with an optimal temperature of 37 °C and retaining 71% of activity at 30 °C, more than twice that we observed for opt.PiXI at 30 °C. We hypothesize that a mesophilic profile of XIs may be a major feature for efficient d-xylose utilization by *S. cerevisiae*. Although the 8054_2 XI we describe here has a thermophilic temperature optimum, we note its activity over a broad thermal range that extends into mesophilic temperatures. This broad range of temperature activity is perhaps not surprising given its origin from the digestive tract of an invertebrate that cannot regulate its body temperature (i.e. is an ectotherm) unlike the endothermic (elephant) habitat of *Piromyces* sp. strain E2 from which opt.PiXI was derived^51^.

To our knowledge, six studies have experimentally compared the XI from *Piromyces* sp. to other XIs. One XI displayed higher affinity but lower specific activity^19^, and another exhibited lower affinity and activity but also lower inhibition by xylitol^8^. A further report identified an enzyme with lower affinity but higher specific activity and another with lower affinity and specific activity^18^, while two additional studies described enzymes with lower specific activity^17,32^. Only one new XI showed higher affinity and specific activity relative to the *Piromyces* sp. XI, which could be further enhanced by evolutionary adaptation^23^. However, as the XI from *Piromyces* sp. was not codon optimized, the comparison is hard to interpret directly. Our study reveals, for the first time, a new XI that exhibits clearly superior kinetics than the XI from *Piromyces* sp. E2 which translates into faster yeast growth and d-xylose consumption in-vivo.

## Conclusions

We have synthesized three putative *xylA* genes from the gut microbiota of a wood-feeding beetle and screened for d-xylose isomerase activity in *S. cerevisiae*. One of these enzymes, 8054_2 XI, expressed actively and showed high identity with XIs from the *Bacteroidia* class of the *Bacteroidetes* phylum. Curiously, the phylogenetic analysis revealed that all XIs from *Bacteroidia* screened in yeast have expressed successfully. The new enzyme showed higher specific activity and affinity for d-xylose than the current gold-standard from *Piromyces* sp., as well as substantially higher relative activity at 30 °C. Superior kinetics of 8054_2 XI correlated with higher specific growth rate and d-xylose consumption. The novel XI represents a highly valuable addition to the *S. cerevisiae* molecular toolbox and shows promise for improved industrial conversion of carbohydrate substrates.

## Methods

### Selection of XI from the metagenome of *O. disjunctus*

Previously published predicted genes and proteins from the metagenome of *O. disjunctus*^20^ were screened against the pfamA-30 database^52^ using HMMER’s hmmscan^53^. Putative metagenome-predicted XI amino acid sequences were aligned together with the XI of *Piromyces* sp. using ClustalW and a phylogenetic tree reconstructed with IQ-TREE using the evolutionary model LG + G4. After the phylogenetic analysis, three sequences were selected and further characterized in the SWISS-MODEL workspace^33^ against the SWISS MODEL template library^54^ and the obtained models evaluated based on amino acid sequence identity, Global Model Quality Estimation (GMQE, evaluated from 0 to 1), and the QMEAN Z-score^55^ (evaluated from − 4 to 0).

### Plasmid constructions

A detailed description of all vectors constructed as a part of this work is available in the form of a collection of Jupyter notebooks^56^. These notebooks contain Python code describing the details of the cloning with the help of the pydna package^57^. These notebooks allow reexamination of the cloning strategies in detail by executing the code. The executable documentation is available in a Git repository (https://github.com/MetabolicEngineeringGroupCBMA/Silva_et_al_2020). The plasmid pLBL3 was used to express candidate d-xylose isomerase genes. The pLBL3 is an expression vector with a *LEU2* auxotrophic marker with both 2µ and pUC origins of replication. The expressed gene is controlled upstream by a TEF1 promoter (the intergenic sequence between MRL1/YPR079W and TEF1/YPR080W) and downstream by the intergenic sequence between YNL095C and RPS7B/YNL096C. Codon-optimized XI genes were synthesized by Integrated DNA Technologies, Inc (Coralville, IA, USA) and were cloned into pLBL3 by in-vivo gap repair between tailed PCR products of individual genes and the plasmid linearized with *Aji*I (Thermo Fisher Scientific Inc, Waltham, MA, USA) (Fig. 1a) in *S. cerevisiae* EBY.VW4000.

The *S. cerevisiae* CEN.PK111-61A was transformed with the pYPK0_XTTRRG vector (Fig. 1b) expressing a partial d-xylose utilization pathway. The vector expresses six different genes, a xylulokinase (*XKS1*), that converts d-xylulose to xylulose 5-phosphate, a d-xylose/glucose facilitator from *Candida intermedia* (Gxf1), and the four genes of the non-oxidative pentose phosphate pathway *TKL1*, *TAL1*, *RPE1* and *RKI1*. *TKL1* codes for a transketolase that convert xylulose-5-phosphate and ribose-5-phosphate to sedoheptulose-7-phosphate and glyceraldehyde-3-phosphate; *TAL1* codes for a transaldolase that converts sedoheptulose 7-phosphate and glyceraldehyde 3-phosphate to erythrose 4-phosphate and fructose 6-phosphate; *RPE1* codes for a d-ribulose-5-phosphate 3-epimerase that converts d-ribulose 5-phosphate to d-xylulose 5-phosphate. *RKI1* codes for a ribose-5-phosphate ketol-isomerase that interconverts ribose 5-phosphate and ribulose 5-phosphate. Yeast transformations were performed as described by the high-efficiency protocol using lithium acetate, ssDNA and polyethylene glycol 3350^58^.

### Strains and cultivation

*Escherichia coli* strain XL1-Blue (Stratagene, La Jolla, CA, USA) was used for routine plasmid preparation. *E. coli* strains were cultivated on lysogeny broth (LB-Lennox) containing 1% (w/v) tryptone (BD biosciences, San Jose, CA, USA), 0.5% (w/v) yeast extract (Panreact AppliChem, Darmstadt, Germany), 0.5% (w/v) sodium chloride and 100 mg L^−1^ ampicillin (Formedium, King's Lynn, UK). *S. cerevisiae* strains were cultivated on complex media containing 2% (w/v) bacto-peptone (BD biosciences, San Jose, CA, USA), 1% (w/v) yeast extract, and 2% (w/v) glucose (YPD), maltose (YPM), or d-xylose (YPX). Yeast strains were also cultivated in defined synthetic complete media (SC) containing 0.67% (w/v) yeast nitrogen base without amino acids (BD, Franklin Lakes, NJ, USA), 0.07% amino acid dropout Kaiser mixture^59^, 50 mM potassium hydrogen phthalate, and 2% (w/v) glucose, maltose or d-xylose. SC media were adjusted to pH 5.5 using sodium hydroxide. Amino acids histidine, uracil and tryptophan were omitted as required for selection of auxotrophic markers. Agar was added to a concentration of 2% (w/v) for solid media. Liquid cultures were incubated with shaking at 200 rpm, yeast at 30 °C and *E. coli* at 37 °C. *S. cerevisiae* strains and plasmids used in this work are listed in Table 2.

**Table 2:** *Saccharomyces cerevisiae* strains and plasmids used in this study.

Strain	Relevant genotype	References
EBY.VW4000	*MATa ura3-52 his3-Δ1 leu2-3,112 trp1-289*	^60^
CEN.PK111-61A	*MATɑ ura3-52 his3-Δ1 leu2-3,112 TRP1*	^61^

Plasmid	Relevant features	References
pYPK0_XTTRRG	*URA3*; *XKS1*, *TAL1*, *TKL1*, *RPE1*, *RKI1*, Gxf1	This work
pLBL3	*LEU2*	This work
pLBL3_8054_2	*LEU2*; 8054_2 *xylA*	This work
pLBL3_15405_2	*LEU2*; 15405_2 *xylA*	This work
pLBL3_1362_6	*LEU2*; 1362_6 *xylA*	This work
pLBL3_opt.PiXI	LEU2; *Piromyces* sp. E2 *xylA*	This work
pLBL3_XR/XDH	LEU2; XR (*XYL1*); XDH (*XYL2*)	This work

### Yeast mating

Each EBY.VW4000 (MATa) strain carrying a pLBL3_XI plasmid was mated with the CEN.PK111-61A/pYPK0_XTTRRG (MATα) strain (Fig. 1c), resulting in diploid yeast strains. Briefly, EBY.VW4000 (pLBL3_XI) and CEN.PK111-61A (pYPK0_XTTRRG) were cultivated in 5 mL YPD medium overnight. Cultures were then diluted in 5 mL YPD medium to an OD600 of 0.2 and grown for at least two generations, to a final OD600 of 0.8–1.2. Cells were harvested by centrifugation and transferred to 1-mL Eppendorf tube. Cell suspensions were washed twice with deionized water and resuspended in 1 mL rich medium. A hundred microliters of each culture were pooled, mixed and incubated overnight at room temperature. The pooled cultures were washed twice in 1 mL deionized water and resuspended in 1 mL water. A hundred microliters of the washed cultures were spread on SC medium. The resulting diploid clones were collected after two days.

### Screening for growth on solid medium

Screening was performed by scoring yeast growth on solid media with d-xylose as the sole, or main, carbon source (Fig. 1d). Recombinant diploids were streaked into 5 mL SC medium containing glucose, lacking leucine, uracil and tryptophan and incubated overnight. Subsequently, 1 mL of culture was washed twice and diluted 1:10 in deionized water. Cell suspensions were incubated at 30 °C overnight and then placed on ice for 2 h. Cultures were serially diluted 1:10 in deionized water. Drops with a volume of 10 µL of dilutions 1, 10^–1^ and 10^–2^ were spotted on YPX and SC media with d-xylose. Plates were incubated for 5 days before scoring growth.

### Liquid culture growth assays

Individual colonies were used to inoculate SC media containing glucose and grown overnight. Yeast cells were washed and transferred to pre-warmed (30 °C) SC medium containing d-xylose at an OD600 of 0.2 and incubated in 50-mL glass tubes for 24 h. Cells were then transferred to 5 mL identical fresh medium in 50-mL shaking-flasks at an OD600 of 0.2 to start measurements. Absorbance was measured using a NanoDrop 1000 spectrophotometer. Cell growth experiments were performed in triplicate.

### d-Xylose consumption

For measuring specific d-xylose consumption, cells from frozen stocks (− 80 °C) were transferred into 50 mL SC media containing glucose and incubated in 500-mL culture flasks for 24 h. Cells were harvested, washed and suspended in 25 mL identical medium containing 4% (w/v) d-xylose, instead of glucose. The inoculation level was 10 g L^−1^ dry cell weight (DCW). Cultivation was performed in 500-mL flasks and samples were taken every 24 h. d-xylose and xylitol concentrations were quantified by high performance liquid chromatography (Hitachi LaChrom Elite) and detection was done by refractive index with an Elite LaChrom L-2490 RI detector (VWR Hitachi). Samples were prepared by adding 125 µL trichloroacetic acid to a final concentration of 10% (v/v) followed by a 2 h incubation on ice. Samples were centrifuged at 4 °C for 15 min and filtered using a 0.22 µm pore hydrophilic PTFE filter. Samples were separated on a Rezex 8 μm ROA-organic acid H^+^(8%) column (Phenomenex) with a mobile phase of 2.5 mM H2SO4, and a flow rate of 0.5 mL min^−1^ at 40 °C. Data was analyzed with the EZChrom Elite 3.3.2 SP2 software. d-xylose consumption experiments were performed in triplicate.

### d-xylose isomerase activity assay

Yeast cells expressing XI enzymes (*Piromyces* sp., 8054_2) were grown overnight in SC media containing glucose. Cultures were diluted in 50 mL same media at an OD600 of 0.3 and incubated for three generations. Cells were harvested, washed twice with water and suspended in 100 mM Tris–HCl buffer pH of 7.5, followed by disruption with glass beads (0.45 mm) using FastPrep FP120 cell disrupter (6.0 oscillations min^−1^ for 20 s). Cell debris was removed by centrifugation at 16.000×*g* for 10 min and the supernatant was conserved on ice. Crude cell extracts were used in the enzyme assays immediately after preparation. Protein concentration was measured by the Bradford assay having bovine serum albumin as standard. All of the enzyme assays were performed in triplicate.

#### Resorcinol method

Resorcinol-based activity assay was performed in this work for determination of optimal temperature of activity of the XI enzymes^62,63^. Cell extracts were diluted in 100 mM Tris–HCl buffer at pH of 7.5. Experiments were carried out by adding 30 µL cell extracts containing 3 mg mL^−1^ total protein to 20 µL test solution (100 mM Tris–HCl at pH of 7.5, 30 mM d-xylose, 0.3 mM MnSO_4_), and then incubated at the testing temperature for an appropriate period of time previously optimized. Sample incubation was carried out into BioRad T100 thermal cycler set at specific temperatures. For color development, 150 µL of a 1:1 mixture (v/v) of solution A (0.05% resorcinol in ethanol) and solution B (0.216 g FeNH_4_(SO_4_)_2_ · 12 H_2_O in 1 L concentrated HCl) were added to the samples followed by incubation at 80 °C for 40 min. The absorbance measurements were determined on the microplate reader SpectraMax Plus 384 (Molecular Devices) at 630 nm.

#### d-Sorbitol dehydrogenase (SDH) method

The SDH method was employed to determine specific activity of the XIs. Assays were performed in mixtures containing 100 mM Tris–HCl (pH value of 7.5), 10 mM MgCl_2_, 0.15 mM NADH, 2 U SDH (Roche Diagnostics, Mannheim, Germany), and 1 mg mL^−1^ cell extract^15^. The reactions started with the addition of the mixture to d-xylose solutions that ranged from 5 to 400 mM. The reactions occurred at 30 °C and were monitored spectrophotometrically (SpectraMax Plus 384, Molecular Devices) at 340 nm through the depletion of NADH resulting from the reduction of xylulose to xylitol by SDH. Kinetic parameters were determined from the interpolation of the experimental data to the Michaelis–Menten curve by the least squares fit method using Graphpad Prism 6.

### Phylogenetic analysis

The amino acid sequences from the metagenome-derived sequences and those from reported studies were aligned using Clustal Omega with default parameters^64^. This software generated a percent identity matrix that was used to compare similarity among the sequences of the enzymes, and an alignment file in “ClustalW” format for phylogenetic tree construction. This file was converted to a usable “Phylip” format^65^. The phylogenetic trees were constructed using PhyML 3.0 on the web server ATGC-Montpellier, using WAG substitution model, mix of NNI/SPR improvements, 8 substitution rate categories, and 1000 bootstrap replicates^66^. The resulting trees were visualized and edited with the on-line software “Interactive Tree of Life”—iTOL v4^67^. GenBank accession numbers of the amino acid sequences used to generate the phylogenetic tree are available in Table S2, Supplementary Information.

## Supplementary Information

Supplementary Information.

## Data Availability

All data generated or analyzed during this study are included in this published article, public repositories, or in the Supplementary Information, as indicated in the manuscript.
