# Supplementary material for: A novel d-xylose isomerase from the gut of the wood feeding beetle Odontotaenius disjunctus efficiently expressed in Saccharomyces cerevisiae
Source: Sci Rep. 2021 Feb 26;11:4766. doi: 10.1038/s41598-021-83937-z (PMC7910561; doi:10.1038/s41598-021-83937-z)
Supplement: Supplementary file 1 — Supplementary Information. [file 41598_2021_83937_MOESM1_ESM.pdf]

Supplementary Information

A novel D-xylose isomerase from the gut of the wood feeding Patent-leather beetle *Odontotaenius disjunctus* efficiently expressed in *Saccharomyces cerevisiae*

Paulo César Silva[a*], Javier A. Ceja-Navarro[bc], Flávio Azevedo[a], Ulas Karaoz[d], Eoin L. Brodie[de*] and Björn Johansson[a*]

[(a)] CBMA - Center of Molecular and Environmental Biology, University of Minho, Campus de Gualtar, Braga, 4710-057, Portugal.

[(b)] Biological Systems and Engineering, Lawrence Berkeley National Laboratory, Berkeley, California, USA.

[(c)] Institute for Biodiversity Science and Sustainability, California Academy of Sciences, San Francisco, California, USA

[(d)] Earth and Environmental Sciences, Lawrence Berkeley National Laboratory, Berkeley, California, USA.

[(e)] Department of Environmental Science, Policy and Management, University of California, Berkeley, California, USA.

*corresponding authors:

Paulo César Silva: pcfernandesdasilva@gmail.com

Eoin L. Brodie: ELBrodie@lbl.gov

Björn Johansson bjorn_johansson@bio.uminho.pt

Table S1. Structure homology parameters between top model sequences and the target sequences.

Target Sequence	Swiss-Model Reference	Description	% identity	GMQE	QMEAN
**8054_2**	4xkm.1.A	Xylose Isomerase	82.45	0.96	-0.45
**15405_2**	1a0e.1.A	Xylose Isomerase	56.68	0.80	-1.29
**1362_6**	1a0e.1.A	Xylose Isomerase	55.92	0.79	-1.60

GMQE is a quality estimation, which combines properties from the target–template alignment and the template search method. The resulting GMQE score is expressed as a number between 0 and 1 - higher number indicates higher reliability. QMEAN around zero indicates good agreement between the model structure.

Table S2. GenBank accession numbers of the amino acid sequences used to generate the phylogenetic tree depicted in the Figure 2 of the manuscript.

#	Source	GenBank	Reference
1	*Thermus thermophilus*	BAA14301	[1]
2	*Piromyces* sp. E2	CAB76571	[2]
3	*Orpinomyces* sp. ukk1	ACA65427	[3]
4	*Clostridium phytofermentans*	BBG40462	[4]
5	Soil – xym1 (unspecified)	AEG75765	[5]
6	Soil – xym2 (unspecified)	AEG75766	[5]
7	*Bacteroides stercoris*	AEK21499	[6]
8	*Ruminococcus flavefaciens*	CAB51938	[7]
9	*Prevotella ruminicola*	AGL34957	[8]
10	*Burkholderia cenocepacia*	CAR57287	[9]

11	*Clostridium cellulovorans*	WP_010074860	[10]
12	*Bacteroides vulgatus*	ABR41556	[11]
13	Bovine rumen (unspecified)	AEL74969	[12]
14	*Sorangium cellulosum*	WP_020464968	[12]
15	Termite gut (unspecified)	HV438106	[13]
16	*Bacteroides thetaiotaomicron* VPI-5482	4XKM_A	[14]
17	*Rhizobium radiobacter*	ACM27770	[15]
18	*Alistipes* sp. HGB5	EFR57390	[11]
19	*Tannerella* sp. 6_1_58FAA_CT1	EHL81684	[11]
20	*Paraprevotella xylaniphila* YIT 11841	EGG54729	[11]
21	*Escherichia coli* MS 175-1	EFJ65453	[16,17]
22	*Bacillus subtilis*	CAA26562	[18]
23	*Actinoplanes missouriensis*	CAA34164	[18]
24	*Lactobacillus pentosus*	AAA25258	[19]
25	*Thermoanaerobacterium thermosulfurigenes*	AAA23285	[20]
26	*Streptomyces rubiginosus*	AAA26838	[21]
27	*Bifidobacterium longum* MG1	AEK21500	[6]
28	*Bacillus licheniformis*	CAB02314	[22]
29	*Paraburkholderia xenovorans*	ABE33371	[22]
30	*Pseudomonas savastanoi pv. Phaseolicola*	AAZ36203	[22]
31	*Robiginitalea biformata*	EAR16136	[22]
32	*Saccharophagus degradans*	ABD81764	[22]
33	*Staphylococcus xylosus*	CAA40824	[22]
34	*Xanthomonas campestris pv. Campestris*	CAP53675	[22]
35	*Salmonella enterica serovar Typhimurium*	ACY90798	[22]
36	*Arabidopsis thaliana*	AAM97134	[22]
37	8054_2 XI **[(a)]**	MT846924	This work

39	1362_6 XI	MT846925	This work
39	15405_2 XI	MT846926	This work

[(a)] The enzyme “8054_2” described in this work was named “8454_2” in the patent applications: U.S. patent application serial number 16/907,108 and International Patent Application PCT/IB2020/055881

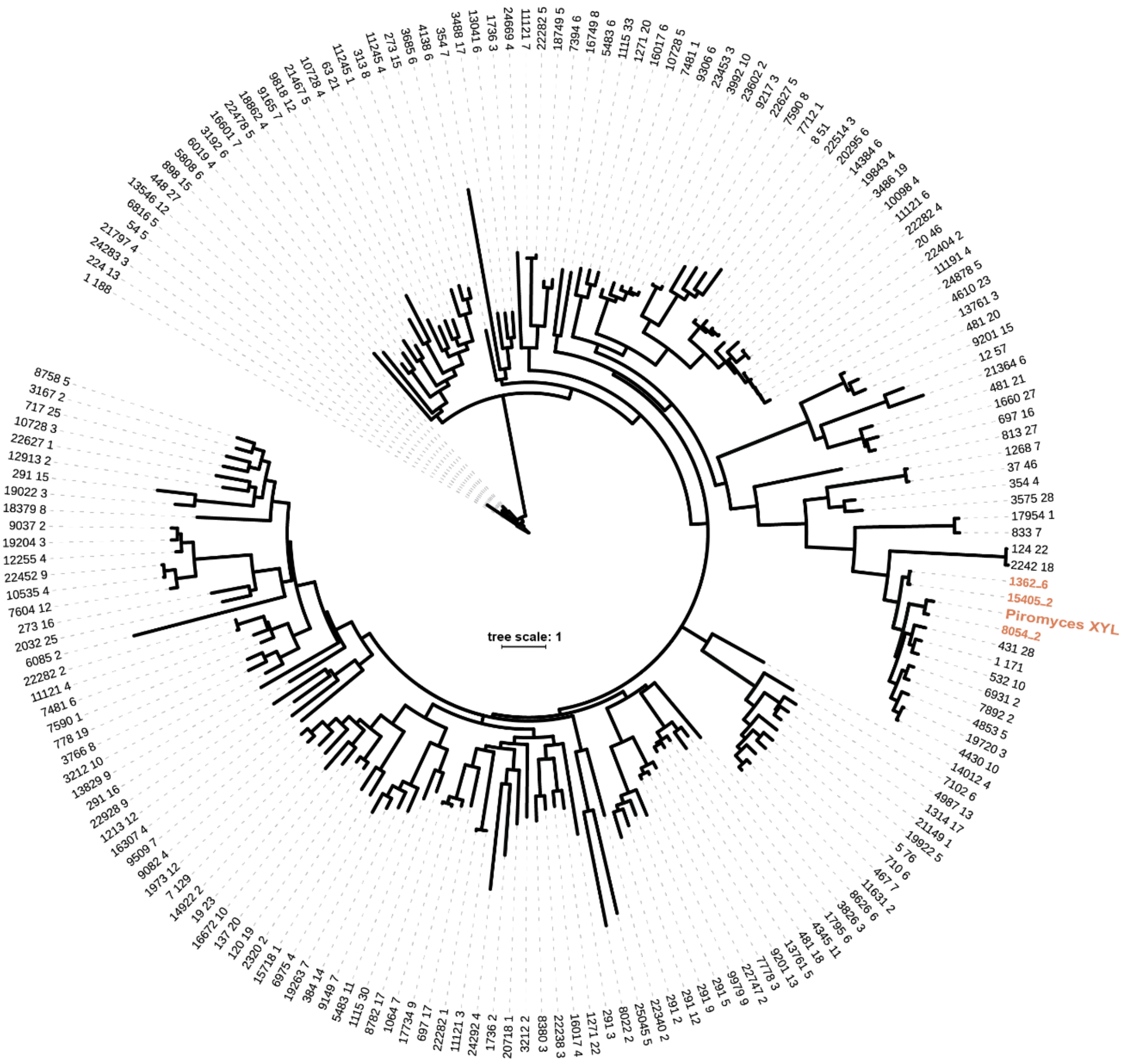

Figure S1. Phylogenetic tree of the amino acid sequences of the putative XI enzymes from the digestive tract of *Odontotaenius disjunctus*. A total of 182 putative XI sequences were detected and phylogenetically placed together with the XI of *Piromyces* sp. E2. Based on their sequence relatedness, the genes 8054_2, 1362_6 and 15405_2 were selected for screening of XI activity in yeast (in orange).

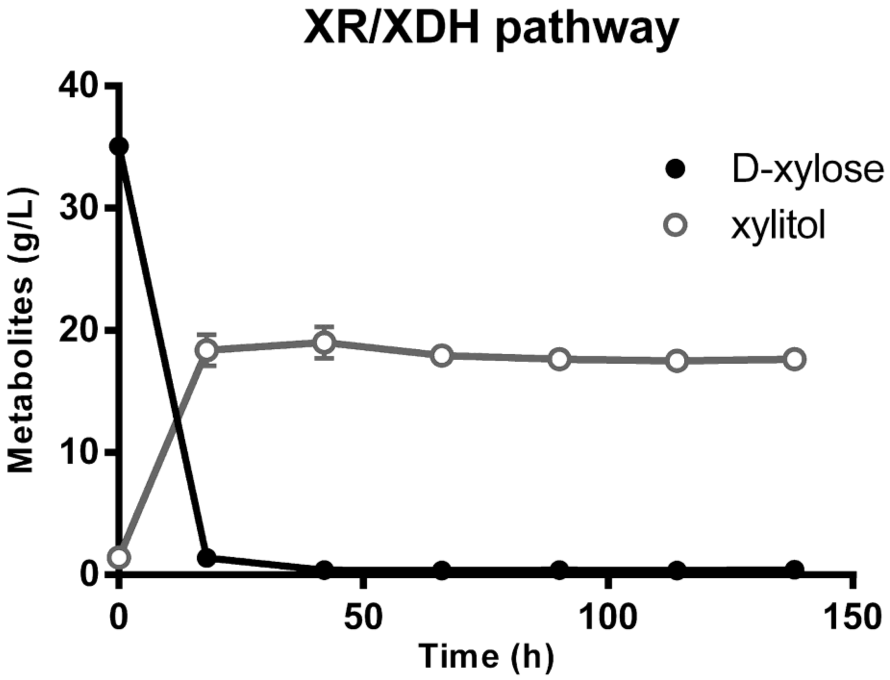

Figure S2. D-xylose consumption by diploid *Saccharomyces cerevisiae* strains expressing XR/XDH enzymes. Cultures were grown under high cell density (10 g/L DCW) in shake flasks on synthetic medium with 4% (w/v) D-xylose as the sole carbon source. Data points represent an average of 3 biological replicates with standard deviation indicated.

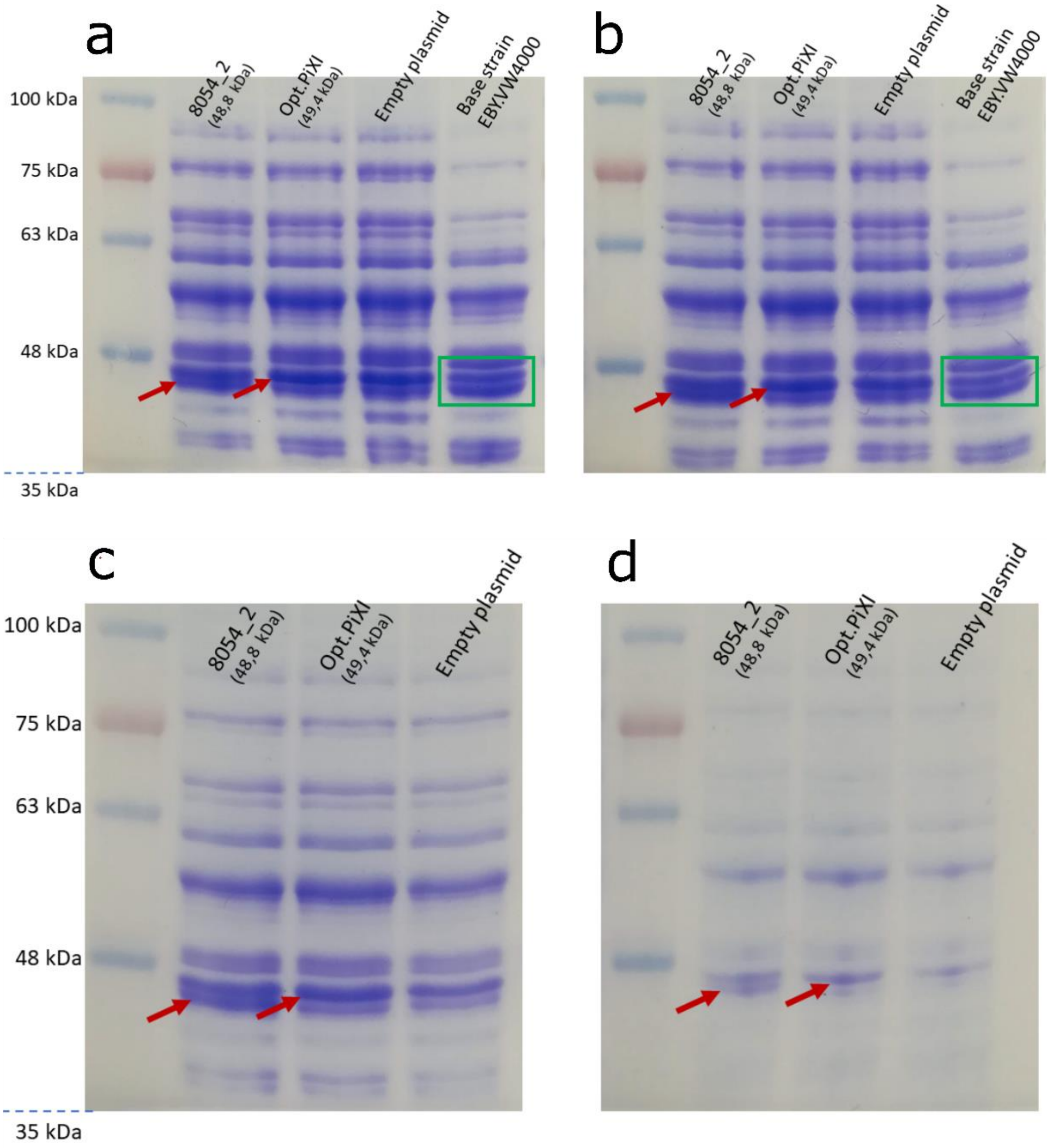

Figure S3. SDS-PAGE of cell extracts of the haploid *Saccharomyces cerevisiae* strain EBY.VW4000 expressing the 8054_2 XI, the codon-optimized XI from *Piromyces* sp. E2 (opt.PiXI), the empty vector, or containing no plasmid. The red arrows indicate the expected protein bands of the XIs and the green rectangles highlight the background proteins produced by the base strain that partly overlap with the XIs. (a) Protein extracts denatured at 60 ºC for 10 minutes; 5 µL sample. (b) Protein extracts denatured at 60 ºC for 20 minutes; 5 µL sample. (c) Protein extracts denatured at 37 ºC for 10 minutes; 5 µL sample. (d) Protein extracts denatured at 37 ºC for 10 minutes; 2 µL sample. The samples were run in 10 % Acrylamide/Bis-acrylamide gels at 100 V using a Mini-PROTEAN II electrophoresis cell (BioRad). The gel was stained with standard Coomassie Blue stain. The molecular weight marker NZYColour Protein Marker II (NZYTech, Lisbon, Portugal) was separated on all gels in the leftmost lane.

References

1. Walfridsson, M. *et al.* Ethanolic fermentation of xylose with Saccharomyces cerevisiae harboring the Thermus thermophilus xylA gene, which expresses an active xylose (glucose) isomerase. *Appl. Environ. Microbiol.* **62**, 4648–4651 (1996).
2. Kuyper, M. *et al.* High-level functional expression of a fungal xylose isomerase: the key to efficient ethanolic fermentation of xylose by Saccharomyces cerevisiae? *FEMS Yeast Res.* **4**, 69–78 (2003).
3. Madhavan, A. *et al.* Xylose isomerase from polycentric fungus Orpinomyces: gene sequencing, cloning, and expression in Saccharomyces cerevisiae for bioconversion of xylose to ethanol. *Appl. Microbiol. Biotechnol.* **82**, 1067–1078 (2009).
4. Seike, T. *et al.* Molecular evolutionary engineering of xylose isomerase to improve its catalytic activity and performance of micro-aerobic glucose/xylose co-fermentation in Saccharomyces cerevisiae. *Biotechnol. Biofuels* **12**, 139 (2019).
5. Parachin, N. S. & Gorwa-Grauslund, M. F. Isolation of xylose isomerases by sequence- and function-based screening from a soil metagenomic library. *Biotechnol. Biofuels* **4**, 9 (2011).
6. Ha, S.-J., Kim, S. R., Choi, J.-H., Park, M. S. & Jin, Y.-S. Xylitol does not inhibit xylose fermentation by engineered Saccharomyces cerevisiae expressing xylA as severely as it inhibits xylose isomerase reaction in vitro. *Appl. Microbiol. Biotechnol.* **92**, 77–84 (2011).
7. Aeling, K. A. *et al.* Co-fermentation of xylose and cellobiose by an engineered Saccharomyces cerevisiae. *J. Ind. Microbiol. Biotechnol.* **39**, 1597–1604 (2012).
8. Hector, R. E., Dien, B. S., Cotta, M. A. & Mertens, J. A. Growth and fermentation of D-xylose by Saccharomyces cerevisiae expressing a novel D-xylose isomerase originating from the bacterium Prevotella ruminicola TC2-24. *Biotechnol. Biofuels*

6, 84 (2013).

9. de Figueiredo Vilela, L. *et al.* Functional expression of Burkholderia cenocepacia xylose isomerase in yeast increases ethanol production from a glucose-xylose blend. *Bioresour. Technol.* **128**, 792–796 (2013).
10. Ota, M. *et al.* Display of Clostridium cellulovorans xylose isomerase on the cell surface of Saccharomyces cerevisiae and its direct application to xylose fermentation. *Biotechnol. Prog.* **29**, 346–351 (2013).
11. Peng, B., Huang, S., Liu, T. & Geng, A. Bacterial xylose isomerases from the mammal gut Bacteroidetes cluster function in Saccharomyces cerevisiae for effective xylose fermentation. *Microb. Cell Fact.* **14**, 70 (2015).
12. Hou, J. *et al.* Characterization and evolution of xylose isomerase screened from the bovine rumen metagenome in Saccharomyces cerevisiae. *J. Biosci. Bioeng.* **121**, 160–165 (2015).
13. Katahira, S. *et al.* Screening and evolution of a novel protist xylose isomerase from the termite Reticulitermes speratus for efficient xylose fermentation in Saccharomyces cerevisiae. *Biotechnol. Biofuels* **10**, 203 (2017).
14. Van Maris, A. J. A. *et al.* Development of efficient xylose fermentation in Saccharomyces cerevisiae: xylose isomerase as a key component. in *Biofuels* 179–204 (Springer, 2007).
15. Kim, D. M. *et al.* Reduction of PDC1 expression in S. cerevisiae with xylose isomerase on xylose medium. *Bioprocess Biosyst. Eng.* **35**, 183–189 (2012).
16. Briggs, K. A., Lancashire, W. E. & Hartley, B. S. Molecular cloning, DNA structure and expression of the Escherichia coli D-xylose isomerase. *EMBO J.* **3**, 611–616 (1984).
17. Sarthy, A. V. *et al.* Expression of the Escherichia coli xylose isomerase gene in Saccharomyces cerevisiae. *Appl. Environ. Microbiol.* **53**, 1996–2000 (1987).
18. Amore, R., Wilhelm, M. & Hollenberg, C. P. The fermentation of xylose — an analysis of the expression of Bacillus and Actinoplanes xylose isomerase genes in

yeast. *Appl. Microbiol. Biotechnol.* **30**, 351–357 (1989).

19. Hallborn, J. Metabolic engineering of saccharomyces cerevisiae: expression of genes involved in pentose metabolism. (Lund University, 1995).
20. Moes, C. J., Pretorius, I. S. & van Zyl, W. H. Cloning and expression of the Clostridium thermosulfurogenes D-xylose isomerase gene (xylA) in Saccharomyces cerevisiae. *Biotechnol. Lett.* **18**, 269–274 (1996).
21. Gárdonyi, M. & Hahn-Hägerdal, B. The Streptomyces rubiginosus xylose isomerase is misfolded when expressed in Saccharomyces cerevisiae. *Enzyme Microb. Technol.* **32**, 252–259 (2003).
22. Brat, D., Boles, E. & Wiedemann, B. Functional expression of a bacterial xylose isomerase in Saccharomyces cerevisiae. *Appl. Environ. Microbiol.* **75**, 2304–2311 (2009).
